# Randomised crossover controlled trial of dietary interventions for glycaemic control when body weight is kept stable

**DOI:** 10.1017/jns.2025.10028

**Published:** 2025-08-29

**Authors:** Maelán Fontes-Villalba, María-Luz Fika-Hernando, Óscar Picazo, Lynda A. Frassetto, Pedro Carrera-Bastos, Ashfaque A. Memon, Giuseppe Lippi, Martina Montagnana, Yvonne Granfeldt, Kristina Sundquist, Jan Sundquist, Tommy Jönsson

**Affiliations:** 1 Center for Primary Health Care Research, Department of Clinical Sciences, Lund University, Malmö, Sweden; 2 Nursing Department, University of Las Palmas de Gran Canaria, Las Palmas de Gran Canaria, Spain; 3 Centro de Estudios Avanzados en Nutrición (CEAN), Cádiz, Spain; 4 Department of Medicine, Division of Nephrology, University of California San Francisco, San Francisco, CA, USA; 5 Department of Neuroscience, Biomedicine and Movement, University Hospital of Verona, Verona, Italy; 6 Department of Food Technology, Engineering and Nutrition, Lund University, Lund, Sweden; 7 University Clinic Primary Care, Skåne University Hospital, Malmö, Sweden

**Keywords:** Blood glucose metabolism, Diabetes mellitus type 2, Diet, Nutrition, Stable body weight, Palaeolithic diet, HbA1c, glycated haemoglobin, LDL, low-density lipoprotein cholesterol, HDL, high-density lipoprotein cholesterol, AUC, area under the curve, OGTT, oral glucose tolerance test

## Abstract

A Palaeolithic diet is an efficacious dietary approach for glycaemic control in type 2 diabetes. Causal mechanisms are body weight loss and glucometabolic effects from differences in included food groups, macronutrient composition, fibre content, and glycaemic load. The aim was to test the hypothesis that characteristic food group differences between a Palaeolithic and a diabetes diet would cause an effect on glycaemic control when weight was kept stable and diets were matched for macronutrient composition, fibre content and glycaemic load. Adult participants with type 2 diabetes and increased waist circumference were instructed to follow two diets, with or without the food groups cereal grain, dairy products, and legumes, during two periods of 4 weeks separated by a 6-week washout period in a random-order crossover design. The Palaeolithic diet included fruit, vegetables, tubers, fish, shellfish, lean meat, nuts, eggs and olive oil, and excluded cereal grains, dairy products and legumes. The diabetes diet included fruit, vegetables, fish, shellfish, lean meat, nuts, eggs, olive oil, and substantial amounts of whole grains, low-fat dairy products and legumes. Dietary energy content was adjusted throughout the study to maintain stable body weight. There were no differences between diets on HbA1c or fructosamine among the 14 participants. Body weight was kept stable, and the two diets were successfully matched for macronutrient composition and glycaemic load but not for fibre content. Characteristic food group differences and the accompanying differences in fibre content between a Palaeolithic and a diabetes diet do not cause an effect on glycaemic control.

## Background

Diabetes results in damage of the heart, blood vessels, eyes, kidneys, and nerves, and is a major global cause of disability and mortality affecting almost one in ten adults.^(1,2)^ Nine out of 10 cases are type 2 diabetes, characterised by hyperglycaemia resulting from ineffective use of insulin by the body, or when the body does not produce enough insulin, or both.^(2)^ A cornerstone in the treatment of type 2 diabetes is optimal glycaemic control.^(3)^ HbA1c is the gold standard measure of glycaemic control used for diagnosis and treatment of type 2 diabetes and reflects glucose exposure over the last 2–3 months.^(4)^ Non-classical methods for assessing glycaemic control include measures that evaluate shorter periods of glucose exposure than HbA1c, such as fructosamine and glycated albumin, which reflect glucose exposure over the last 2–4 and 2–3 weeks, respectively.^(4)^

A network meta-analysis found that a Palaeolithic diet is an efficacious dietary approach for glycaemic control in type 2 diabetes.^(3)^ Underlying causal mechanisms discussed include body weight loss and glucometabolic effects resulting from dietary differences in included food groups, macronutrient composition, fibre content, and glycaemic load.^(3,5)^ Underlining the importance of body weight loss, we previously found no difference in glycaemic control in a randomised controlled trial comparing a Palaeolithic diet with a Mediterranean-like diet where there was also no difference in body weight loss.^(6)^ In contrast, we found greater improvement in glycaemic control (as measured by HbA1c) from a Palaeolithic diet compared with a diabetes diet in a randomised controlled crossover trial where there was also greater body weight loss with the Palaeolithic diet.^(7)^ In both studies, the content of the diets differed regarding the characteristic food groups cereal grains, dairy products, and legumes, which by design were excluded from the Palaeolithic diet and included in the control diets.^(6,7)^ The diets also differed in resulting macronutrient composition, fibre content, and glycaemic load.^(6,7)^ Both similar and contradictory results were found in two other intervention studies on the Palaeolithic diet.^(8,9)^ One study reported similar macronutrient composition but did not report fibre intake or glycaemic load.^(8)^

These findings raise interesting questions regarding causal mechanisms. Is effect of the Palaeolithic diet on glycaemic control entirely mediated by accompanying weight loss, or can it also be attributed to glucometabolic effects resulting from differences between a Palaeolithic and a diabetes diet in included food groups, macronutrient composition, fibre content, and glycaemic load?

In our previous studies, a Palaeolithic diet resulted in a significantly higher intake of fruits and vegetables compared with control diets.^(6,7)^ Higher intakes of fruits and vegetables have been shown to have beneficial effects on insulin sensitivity as shown in both intervention^(10)^ and observational^(11)^ studies, which can lead to a reduced risk of type 2 diabetes according to observational studies.^(11–13)^ Increased consumption of fruits and vegetables has also been shown to reduce dietary intake of advanced glycated end products (AGEs), as indicated by a practical dietary guide.^(14)^ AGEs are produced in the body through a nonenzymatic reaction between reducing sugars and proteins, lipids or nucleic acids, as part of normal metabolism. However, their production can be increased by certain cooking methods, particularly those involving high temperatures and low humidity.^(14)^ The pathologic effects of AGEs are attributed to their ability to impair protein structure and function and to their pro-oxidant and pro-inflammatory properties, which can impair protein structure and function as well as disrupt cell surface receptors signalling. Dietary intake of AGEs has been associated with insulin resistance which might contribute to increased risk of type 2 diabetes as indicated by a systematic review and meta-analysis of randomised controlled trials.^(15)^ Furthermore, non-Palaeolithic dietary constituents such as cereal grain proteins could cause leptin or insulin resistance,^(16)^ as congruently assessed in an in vitro study showing that digested wheat gluten proteins inhibit leptin binding to its receptor in a dose-dependent manner at clinically relevant concentrations.^(17,18)^ Leptin—a hormone primarily secreted by the adipose tissue—reduces appetite when binding to its receptor in the brain.^(19,20)^ In most people with obesity, leptin’s regulatory effects are impaired, a condition termed leptin resistance, which can lead to increased circulating leptin levels.^(21)^ In addition, leptin resistance has been suggested to be involved in the pathogenesis of type 2 diabetes.^(22)^ Consistently, also in our previous studies, a Palaeolithic diet resulted in lower leptin levels compared to control diets,^(18,23,24)^ and the strongest correlation between change in leptin and dietary variables was with cereal grain intake.^(23)^ Based on these food group-related glucometabolic effects, we hypothesised that characteristic food group differences between a Palaeolithic and a diabetes diet would cause an effect on glycaemic control beyond the effects resulting from accompanying differences in body weight change, macronutrient composition, fibre content, and glycaemic load.

The aim of this study was to test this hypothesis by comparing the effects on glycaemic control of two diets with or without the food groups cereal grain, dairy products, and legumes while keeping participants’ body weight stable and matching both diets for macronutrient composition, fibre content, and glycaemic load.^(5,25)^

## Methods

The Ethics Committee of Clinical Investigation (CEIC) of the Hospital Universitario de Gran Canaria, Doctor Negrín (Code CEIC Negrín: 130030) approved the study protocol, which adhered to the Declaration of Helsinki, and all participants gave informed consent. The fully detailed protocol of this trial has been published.^(25)^ Therefore, here we will briefly describe the methods and refer the reader to the study protocol for further details.

### Procedures

The study was an open-label randomised crossover dietary intervention trial in participants with type 2 diabetes and increased waist circumference who were instructed to follow diet A and B during two 4-week periods separated by a 6-week washout period. Diet A included fruit, vegetables, fish, shellfish, lean meat, nuts, eggs, and olive oil, as well as substantial amounts of whole grains, low-fat dairy products, and legumes. Diet B included fruit, vegetables, fish, shellfish, lean meat, nuts, eggs, and olive oil and excluded cereal grains, dairy products, and legumes, which were largely replaced by root vegetables (including potatoes), vegetables, fruit, and lean meat, and slightly more fish and nuts. Salt intake was lower in diet B, but macronutrient composition, fibre content, and glycaemic load were matched when designing both diets. To decrease the risk of bias, great care was given to present both study diets as equally favourable. To minimise the risk of bias associated with naming a specific dietary pattern, the term ‘Palaeolithic diet’ was avoided. Instead, participants were informed that the study aimed to compare two healthy diets (diet A and diet B), as it was not yet known which, if any, was superior. However, for clarity and simplicity in this article, we refer to ‘diet A’ as the ‘diabetes diet’ and ‘diet B’ as the ‘Palaeolithic diet’.

Both diets were classified as ‘very healthy’ using the Spanish validated nutritional software DIAL (Alce Ingeniería, Madrid, Spain, 2004),^(26)^ and were in accordance with official Spanish dietary recommendations for people with type 2 diabetes regarding macronutrient composition, fibre content, minerals, and vitamins (Tables 1 and 2 in the study protocol). There were no official Spanish dietary recommendations regarding glycaemic load. Lunch was provided daily in a hospital kitchen to improve compliance. Breakfast, morning and afternoon snacks, and dinner were eaten at home by the participants according to detailed dietary instructions with specified weights for each food item. A detailed description of each of the daily meals proposed for 1 week for both diets—including specific weights—is included in Additional file 3 of the study protocol.^(25)^ Advice about regular physical activity was given equally to all participants. Specifically, they were recommended not to change their physical activity during the study period.^(25)^

**Table 1.** Baseline values and relative change during diet

Variable			Relative change during diet (%)						*P*
	Variable		Diabetes diet		Palaeolithic diet		Difference		
Female/male, *n (%)*	7 (50%)/7 (50%)								
Age, years *M (SD)*	64	(7)							
Diabetes duration, years *M (SD)*	8	(5)							
Height, cm *M (SD)*	164	(7)							
BMI, kg/m^2^ *M (SD)*	32	(5)							
Fructosamine, μmol/l *M (SD) Mdn* (Range) *M* [95% CI]	317	(268–476)	–13	(8)^d^	–11	(9)^d^	–3	[–10, 4]	.40
HbA1c, % *M (SD) Mdn* (Range) *Mdn* [95% CI]	7.4	(1)	0.6	(–25–5)	–1.1	(7)	2	[–6, 5]	.80
Glycated albumin, % *M (SD) M* [95% CI]^a^	34	(9)	–11	(15)^d^	–11	(13)^d^	1	[–12, 14]	.90
Glucometer, mg/dl *M (SD) Mdn* (Range) *M* [95% CI]	133	(123–224)	0	(29)	–14	(17)^d^	14	[–8, 36]	.20
Fasting glucose, mmol/l *M (SD) M* [95% CI]	8	(2)	–5	(27)	–8	(22)	3	[–16, 22]	.70
Fasting insulin, ng/ml *M (SD) M* [95% CI]	0.9	(1)	–12	[–32, 12]^c^	–1	(34)	–6	[–37, 25]	.70
Total AUC glucose_0–120_, mmol/l/min *M (SD) M* [95% CI]	1571	(263)	0	(22)	2	(18)	–2	[–20, 17]	.80
Total AUC insulin_0–120_, ng/ml/min *M (SD) M* [95% CI]^e^	186	(66)	7	(36)	2	(42)	5	[–8, 17]	.40
Adiponectin, mg/ml *M (SD) M* [95% CI]	3.2	(2)	–3	(27)	–11	(23)	7	[–8, 22]	.30
Leptin, ng/ml *M (SD) M* [95% CI]	7.5	(6)	–9	(32)	–11	(34)	2	[–18, 22]	.80
Total cholesterol, mmol/l *M (SD) Mdn* [95% CI]	4.2	(1)	–7	[–17, 4]^c^	–15	(14)^d^	7	[–4, 18]	.30
LDL, mmol/l *M (SD) Mdn* (Range) *Mdn* [95% CI]	2.3	(0)	–17	(–36–67)	–26	(16)^d^	11	[–5, 26]	.20
HDL, mmol/l *M (SD) Mdn* [95% CI]	1.2	(0)	–13	(14)^d^	–6	(16)	–5	[–16, 3]	.30
Triglyceride, mmol/l *M (SD) M* [95% CI]	1.6	(1)	26	(39)^d^	–1	(23)	27	[–2, 56]	.10
C-reactive protein, mg/l *M (SD) Mdn* [95% CI]^e^	3.2	(3)	–17	[–47, 20]^c^	30	[–35,125]^c^	–14	[–177, 36]	.60
Body weight, kg *M (SD) M* [95% CI]	86	(14)	–0.57	(1)	–1.07	(2)	0	[–1, 2]	.30
Waist circumference, cm *M (SD) M* [95% CI]	108	(11)	–1	(2)	–1	(2)	0	[–1, 2]	.60
Hip circumference, cm *M (SD) M* [95% CI]	108	(10)	–1	(2)	–1	(2)	0	[–3, 2]	.80
Sagittal abdominal height, cm *M (SD) M* [95% CI]^*a*^	26	(3)	1	(3)	0	(3)	1	[–1, 3]	.30
Skinfold biceps, mm *M (SD) M* [95% CI]^bf^	22	(12)	3	(12)	–18	(14)^d^	21	[6, 37]	.01
Skinfold triceps, mm *M (SD) M* [95% CI]	31	(12)	–1	(23)	–6	(16)	6	[–8, 19]	.40
Skinfold suprailiac, mm *M (SD) M* [95% CI]	34	(7)	–7	(10)^d^	–2	(12)	–4	[–15, 7]	.40
Skinfold subscapular, mm *M (SD) M* [95% CI]	38	(10)	–1	(15)	–2	(7)	1	[–11, 12]	.90
Systolic Blood Pressure, mmHg *M (SD) M* [95% CI]	133	(8)	3	[–2, 8]^c^	2	(17)	2	[–10, 13]	.70
Diastolic Blood Pressure, mmHg *M (SD) M* [95% CI]	77	(5)	–1	(8)	4	(17)	–5	[–15, 6]	.40
Total body fat, kg *M (SD) M* [95% CI]	34	(9)	0	(6)	–1	(6)	1	[–3, 5]	.70
Visceral fat, kg *M (SD) Mdn* (Range) *M* [95% CI]	14	(3)	–3	(5)^d^	0	(–8–8)	–3	[–7, 1]	.10
Muscle mass, kg *M (SD) M* [95% CI]	53	(9)	0	(2)	–1	(4)	1	[–2, 3]	.60
Bone mass, kg *M (SD) Mdn* (Range) *Mdn* [95% CI]	3	(0)	0	(–4–5)	–1	(3)	1	[–2, 3]	.50
Body water, % *M (SD) M* [95% CI]	48	(7)	0	(2)	0	(3)	0	[–2, 2]	.90
Resting Energy Expenditure, MJ *M (SD) M* [95% CI]	7.0	(1)	0	(2)	–1	(3)	1	[–1, 3]	.50

BMI, body mass index; HbA1c, glycated haemoglobin; AUC, area under curve at oral glucose tolerance test (OGTT); LDL, low-density lipoprotein cholesterol; HDL, high-density lipoprotein cholesterol.

a Period effect.

b Carry-over effect, only data from first intervention period used.

c Geometric mean.

d *P* < .05 for mean change during diet.

e *P* < .05 for mean difference between diets at baseline.

f *P* < .05 for mean difference between diets in change during diet.

Baseline characteristics and outcome measures of participants, relative change during each diet and the difference between relative changes between a Palaeolithic and a diabetes diet after 4 weeks. Normally distributed variables are presented as *M* (*SD*). Transformed variables with normal distribution are presented as *Mdn* [95% CI]. Variables not normally distributed, neither before nor after transformation, are presented as *Mdn* (Range).

**Table 2.** Daily dietary intake from food groups at baseline

Variable	Diabetes diet (N = 10)		Palaeolithic diet (N = 13)		Difference (N = 9)	
Total weight, kg *M* (*SD*)	2.1	(0.5)	2.0	(0.4)	0.1	[–0.3, 0.5]
Total energy, MJ *M* (*SD*) *Mdn* [95% CI]	8.6	(2.6)	9.1	(2.0)	–1.0	[–1.7, 2.6]
Vegetables, g *M* (*SD*) *M* [95% CI]	278	[223, 348]^a^	288	(84)	3	[–75, 82]
Vegetables, kJ *M* (*SD*) *Mdn* [95% CI]	438	(235)	416	(164)	–10	[–104, 297]
Fruits, g M (*SD*) *M* [95% CI]	450	[309, 655]^a^	517	(378)	–100	[–414, 213]
Fruits, kJ *M* (*SD*) *M* [95% CI]	1174	(658)	909	[577, 1431]^a^	–134	[–803, 535]
Meats and meat derived products, g *M* (*SD*) *M* [95% CI]	135	(81)	126	(90)	33	[–20, 85]
Meats and meat derived products, kJ *M* (*SD*) *M* [95% CI]	915	(678)	822	(458)	201	[–324, 726]
Fish and fish derived foods, g *M* (*SD*) *Mdn* [95% CI]	129	(106)	96	(79)	–24	[–46, 36]
Fish and fish derived foods, kJ *M* (*SD*) *Mdn* (Range) M [95% CI]	460	(365)	254	(0–1482)	–70	[–260, 120]
Eggs, g *M* (*SD*) *Mdn* [95% CI]	34	(18)	37	(26)	4	[–24, 11]
Eggs, kJ *M* (*SD*) *M* [95% CI]	196	(109)	216	(153)	–26	[–118, 66]
Cereals, g *M* (*SD*) *M* [95% CI]	195	(74)	223	(72)	–15	[–69, 39]
Cereals, kJ *M* (*SD*) *M* [95% CI]	2528	(1018)	2851	(1021)	–70	[–844, 704]
Legumes, g *M* (*SD*) *Mdn* [95% CI]	46	(46)	31	(29)	–6	[–18, 29]
Legumes, kJ *M* (*SD*) *M* [95% CI] *Mdn* [95% CI]	252	[71, 869]^a^	400	(415)	–111	[–302, 327]
Dairy products, g *M* (*SD*) *Mdn* [95% CI]	333	(267)	380	(151)	–87	[–207, 236]
Dairy products, kJ *M* (*SD*) *Mdn* [95% CI]	1077	(908)	1165	(466)	–199	[–573, 931]
Sugars and cakes, g *Mdn* (Range) *M* [95% CI]	0	(0–10)	1	(0–54)	–2	[–8, 4]
Sugars and cakes, kJ *Mdn* (Range) *M* [95% CI]	1	(0–206)	21	(0–917)	–22	[–125, 82]
Oils and fats, g *M* (*SD*) *M* [95% CI]	25	(15)	30	(18)	1	[–13, 14]
Oils and fats, kJ *M* (*SD*) *M* [95% CI]	933	(550)	1109	(667)	31	[–470, 532]
Drinks, g *M* (*SD*) *Mdn* [95% CI]	385	(299)	247	(218	25	[–113, 628]
Drinks, kJ *Mdn* (Range) *Mdn* [95% CI]	41	(0–636)	0	(0–955)	0	[–15, 35]
Pre-cooked foods, g *Mdn* (Range) *M* [95% CI]	0	(0–2)	0	(0–150)	0	[–1, 1]
Pre-cooked foods, kJ *Mdn* (Range) *M* [95% CI]	3	(0–21)	5	(0–917)	–1	[–11, 9]
Snacks, g *Mdn* (Range) *M* [95% CI]	2	[0, 6]^a^	0	(0–13)	1	[–5, 7]
Snacks, kJ *M* [95% CI] *Mdn* (Range) M [95% CI]	12	[2, 34]^a^	0	(0–36)	10	[–19, 39]
Sauces and dressings, g *M* [95% CI] *Mdn* [95% CI]	2	[1, 4]^a^	5	[2, 11]^a^	–1	[–3, 2]
Sauces and dressings, kJ *Mdn* (Range) *M* [95% CI]	3	(0–86)	4	(0–825)	0	[–3, 2]

a Geometric mean.

Average food eaten per day by weight and energy at baseline. Estimated from 4-day weighed food records. Normally distributed variables are presented as *M* (*SD*). Transformed variables with normal distribution are presented as *Mdn* [95% CI]. Variables not normally distributed, neither before nor after transformation, are presented as *Mdn* (Range).

The predefined primary outcome was HbA1C. Two weeks after randomisation and the concurrent start of the study, we added fructosamine as a primary outcome, reasoning that its shorter evaluation period of glucose exposure over the last month would improve the study by being a better fit to the 1-month diet interventions than HbA1c, which reflects glucose exposure over 2–3 months.^(4)^ For the same reason, after the study completion, we also added glycated albumin as a secondary outcome.^(4)^ Predefined secondary outcomes were fasting plasma glucose, total cholesterol, low-density lipoprotein cholesterol (LDL), high-density lipoprotein cholesterol (HDL), triglycerides, high-sensitivity C-reactive protein, blood pressure, area under the curve (AUC) for glucose during an oral glucose tolerance test (OGTT), anthropometric measurements, satiety quotient, and change in medication. We included adult (>18 years old) males and females with increased waist circumference (≥ 80 cm for women and ≥ 94 cm for men) and a medical diagnosis of type 2 diabetes (certified in writing by their physician), with or without medication (including insulin treatment), and with stable weight for 3 months prior to the start of the study. Food intake during the study was assessed using participants’ 4-day weighed food records (including 1 weekend day), with weighing of each food item on an electronic weighing scale (that could be set to zero).

### Statistics

The SPSS statistical computer package (version 28.0; IBM Corporation, Armonk, NY, USA) was used for all statistical analyses. A pre-study power calculation showed that to detect, with 80% power and at a significance level of 5%, a 0.6 percentage point (4 mmol/mol) difference between diets in change in HbA1c, 13 participants were estimated to be required. Similarly, to detect a 26 μmol/l difference in change in fructosamine, 12 participants would be required. Normal Q–Q plot assessments and the Shapiro-Wilk test were performed to examine whether variables were normally distributed. Normally distributed variables are presented as means with standard deviations. When variables were not normally distributed, logarithmic transformation was applied to assess if normal distribution could be achieved. Transformed variables with a normal distribution are presented as geometric means with 95% confidence intervals. Variables not normally distributed neither before nor after transformation are presented as medians with ranges. Mean comparisons between participants were performed using a two-tailed unpaired *t*-test or a Mann-Whitney *U* test, as appropriate. Mean comparisons within participants were performed using a two-tailed paired *t*-test or a Wilcoxon signed-ranks test, as appropriate. Period effect was tested for by comparing the means of the differences in change during diets between participants starting with the Palaeolithic diet or diabetes diet. Carry-over effect was tested for by comparing the means of the mean change during diets between participants starting with the Palaeolithic diet or diabetes diets. All statistical tests were two-sided, and statistical significance was set at *P* < .05.

## Results

**Figure 1.**
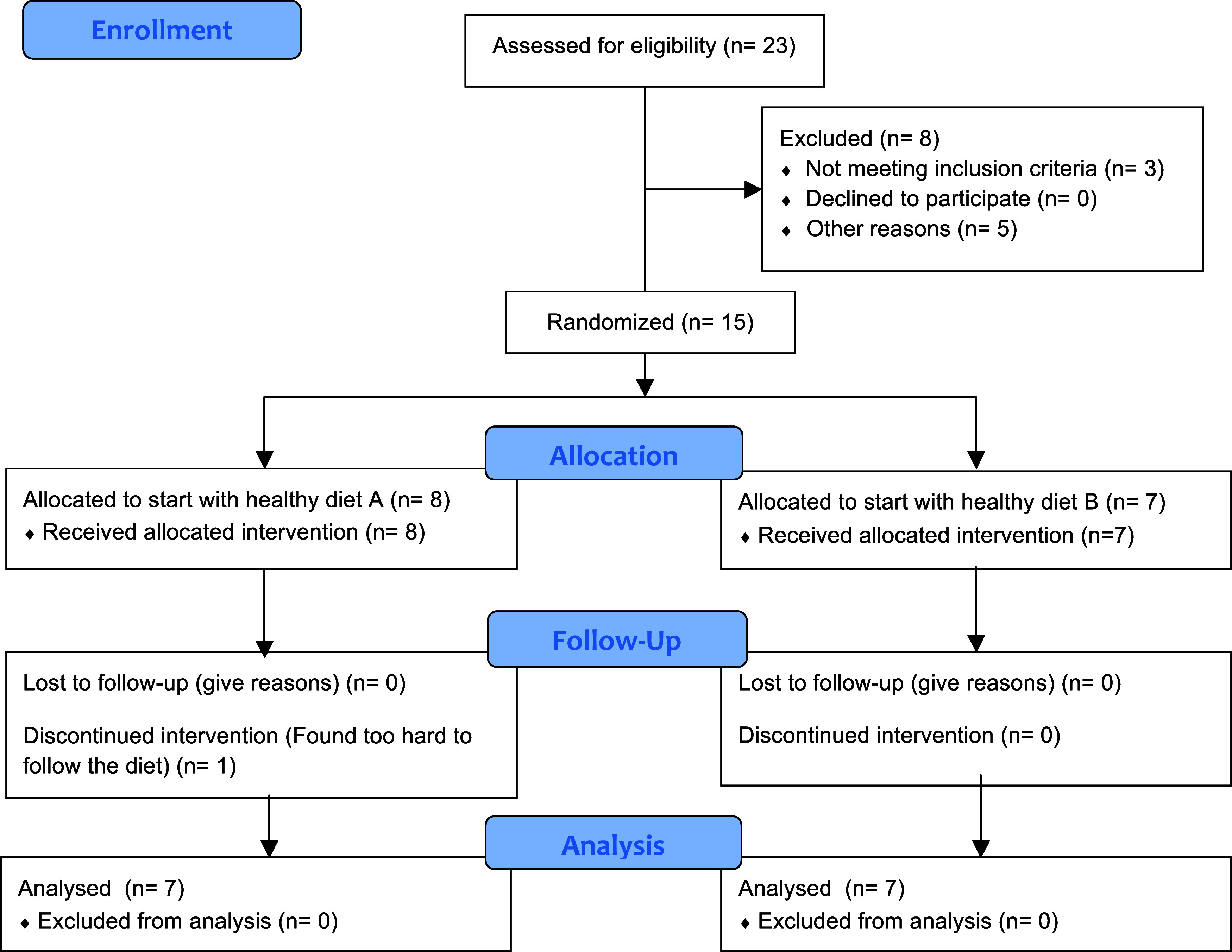
CONSORT 2010 flow diagram.

Recruitment started in August 2013 and finished in November 2013. A total of 23 participants were assessed for eligibility, and eight were excluded. Fifteen participants met the inclusion criteria and were randomised to start the trial. One participant randomised to start with the diabetes diet discontinued the intervention after the first day because the participant found the protocol too difficult to follow. All participants started the trial at the same time. The first diet intervention started on Monday, 18^th^ November 2013, and finished on Sunday, 15^th^ of December 2013. The second diet intervention started on Monday, 27^th^ January 2014, and finished on Sunday, 23^rd^ of February 2014. Fourteen participants (seven women and seven men; seven randomised to start with the diabetes diet and seven randomised to start with the Palaeolithic diet) completed the trial and were analysed per protocol for the primary outcome (Figure 1, Table 1). There were no reported harms to the participants derived from the interventions at any time point during the trial.

There were no differences between the two diets in relative change for the primary outcomes HbA1c or fructosamine (Table 1).

One measurement was missing on one occasion for one participant each for subscapular skinfold, LDL, fasting insulin, and HbA1c. Regarding glucose and insulin during the OGTT, around one in six measurements were missing due to difficulties in drawing blood from several participants, resulting in missing AUC values for glucose in five participants and for insulin in six participants. Blood pressure medication increased during the second intervention (Palaeolithic diet) for one participant and at the start of the second intervention (Palaeolithic diet) for another participant; all blood pressure values were disregarded for these two participants. For all other reported outcome variables, all measurements were completed and analysed in all participants who completed the trial.

During the diabetes diet there was a relative decrease in HDL, suprailiac skinfold, and visceral fat, and a relative increase in triglycerides. During the Palaeolithic diet there was a relative decrease in total cholesterol, LDL, and biceps skinfold (Table 1). There was a significant difference between diets in relative change during a diet for biceps skinfold (+3% *SD* 12% and –18% *SD* 14% for the diabetes and Palaeolithic diets, respectively, *P* = .01, Table 1). There were no other differences between diets in relative change during a diet for any reported outcome variable. There were also no changes in insulin or other diabetes medications, nor in medications for lowering blood lipids or regulating body metabolism (thyroxine) during the study.

There were period effects in glycated albumin and sagittal abdominal height, with a smaller relative decrease for all participants during the first intervention period compared to the second in glycated albumin (–3% *SD* 12% and –19% *SD* 10%, respectively. *P* < .01), and a relative increase for all participants during the first intervention period compared to a relative decrease during the second in sagittal abdominal height (1% *SD* 3% and –1% *SD* 3%, respectively. *P* = .048). There was a carry-over effect in biceps skinfold (*P* = .04), and therefore only data from the first intervention period were used to test for differences in biceps skinfold between diets.

There were no baseline differences in daily food group or nutrient intake when participants started either the diabetes or the Palaeolithic diet (Tables 2–3). At the end of the interventions, daily food group intake by energy was higher in the diabetes diet for eggs, cereal grains, legumes, dairy products, oils and fats, and sauces and dressing, and lower for total energy, vegetables, fruits, meat, fish, sugars and cakes and snacks compared with the Palaeolithic diet (Table 4). When daily food group intake was analysed by weight at the end of the interventions, the diabetes diet was higher in eggs, cereal grains, legumes, dairy products, oils and fats, and drinks and lower in total food weight, vegetables, fruits, meat, fish, sugars and cakes, and snacks compared with the Palaeolithic diet (Table 4). In terms of daily dietary nutrient intake at the end of the interventions, the diabetes diet was higher in calcium, sodium, vitamin B1 and B2, myristic and stearic acids, retinol, zinc and selenium, and lower in protein (by weight, although not by energy percentage), fibre, polyunsaturated fatty acids, vitamin A, folic acid, vitamins C and B12, glucose, fructose, saccharose, palmitoleic, linoleic, alpha-linolenic and arachidonic acids, niacin, folate, beta carotene, vitamin E, tocopherols and potassium compared with the Palaeolithic diet (Table 5). There were no differences between diets in adjustments of recommended daily dietary energy intake during a diet to maintain a stable body weight (Supplementary Table 1).

**Table 3.** Daily dietary nutrient intake at baseline

Variable	Diabetes diet (N = 10)		Palaeolithic diet (N = 13)		Difference (N = 9)	
Total weight, kg *M* (*SD*) *M* [95% CI]	2.1	(0.5)	2.0	(0.4)	0.1	[–0.3, 0.5]
Total energy, MJ *M* (*SD*) *Mdn* [95% CI]	8.6	(2.6)	9.1	(2.0)	–1	[–1.7, 2.6]
Protein, g *M* (*SD*) *M* [95% CI]	94	(31)	91	(16)	5	[–14, 23]
Protein energy percent, E% *M* (*SD*) *M* [95% CI]	18	(2)	17	(2)	1	[–1, 2]
Carbohydrates, g *M* (*SD*) *M* [95% CI]	226	(67)	239	(55)	–4	[–63, 54]
Carbohydrates energy percent, E% *M* (*SD*) *M* [95% CI]	48	(6)	48	(8)	–2	[–8, 4]
Fibre, g *M* (*SD*) *M* [95% CI]	31	(10)	32	(11)	–2	[–15, 11]
Fat, g *M* (*SD*) *M* [95% CI]	75	(27)	84	(31)	3	[–17, 23]
Fat energy percent, E% *M* (*SD*) *M* [95% CI]	33	(5)	34	(7)	1	[–4, 7]
Cholesterol, mg *M* (*SD*) *M* [95% CI]	321	(69)	336	(109)	13	[–73, 99]
Saturated fatty acids, g *M* (*SD*) *M* [95% CI]	22	(9)	25	(12)	3	[–4, 9]
Monounsaturated fatty acids, g *M* (*SD*) *M* [95% CI]	34	(14)	39	(15)	2	[–9, 13]
Polyunsaturated fatty acids, g *M* (*SD*) *M* [95% CI]	10	(4)	12	(4)	–1	[–3, 1]
Calcium, mg *M* (*SD*) *Mdn* [95% CI]	890	(448)	986	(291)	–281	[–360, 363]
Iron, mg *M* (*SD*) *M* [95% CI]	18	(5)	16	(4)	1	[–3, 5]
Sodium, mg *M* (*SD*) *M* [95% CI]	1936	[1406, 2665]^a^	2309	(739)	73	[–641, 787]
Vitamin A, µg *M* (*SD*) *Mdn* (Range) *M* [95% CI] *Mdn* [95% CI]	1280	(656–18227)	1158	[985, 1361]^a^	106	[–38, 2476]
Vitamin B1, mg *M* (*SD*) *M* [95% CI]	1.7	(0.6)	1.6	(0.5)	–0.1	[–0.5, 0.3]
Vitamin B2, mg *M* (*SD*) *M* [95% CI]	2.1	(0.7)	2.0	(0.4)	0.1	[–0.4, 0.6]
Vitamin C, mg *M* (*SD*) *M* [95% CI] *Mdn* [95% CI]	178	(86)	166	[125, 221]^a^	–19	[103, 140]
Vitamin B12, µg *M* (*SD*) *M* [95% CI] *Mdn* [95% CI]	7.2	[3.5, 14.5]^a^	5.7	(2.8)	1.4	[–0.6, 17.5]
Water, g *M* (*SD*) *M* [95% CI]	1450	(414)	1351	(280)	119	[–180, 418]
Alcohol, g *M* (*SD*) *Mdn* (Range) *Mdn* [95% CI]	0.2	(0.0–18.1)	0.0	(0.0–17.2)	0.0	[–1.3, 0.3]
Glucose, g *M* (*SD*) *M* [95% CI]	14	(69	14	[10, 19]^a^	–1	[–6, 4]
Fructose, g *M* (*SD*) *M* [95% CI]	20	(89	18	[13, 27]^a^	–3	[–13, 7]
Saccharose, g *M* (*SD*) *M* [95% CI]	22	(10	19	(9)	2	[–6, 11]
Myristic acid, g *M* (*SD*) *M* [95% CI]	2.0	(1.1)	2.1	(1.0)	0.2	[–0.7, 1.2]
Palmitic acid, g *M* (*SD*) *M* [95% CI]	12	(59	13	(4)	1	[–2, 5]
Stearic acid, g *M* (*SD*) *M* [95% CI]	4.2	[3.0, 5.8]^a^	4.7	(1.7)	0.6	[–1.1, 2.3]
Palmitoleic acid, g *M* (*SD*) *Mdn* (Range) *M* [95% CI]	1.4	(0.8–2.0)	1.5	(0.5)	0.1	[–0.1, 0.4]
Oleic acid, g *M* (*SD*) *M* [95% CI]	32	(13)	36	(14)	2	[–9, 13]
Linoleic acid, g *M* (*SD*) *M* [95% CI]	7.8	(2.9)	9.8	(3.3)	–1.3	[–3.1, 0.4]
Alpha-linolenic acid, g *M* (*SD*) *M* [95% CI]	1.1	(0.5)	1.2	(0.4)	–0.1	[–0.4, 0.2]
Arachidonic acid, g *M* (*SD*) *M* [95% CI] *Mdn* [95% CI]	0.2	(0.1)	0.1	[0.1, 0.2]^a^	0.0	[0.0, 0.1]
Niacin, mg *M* (*SD*) *M* [95% CI]	40	(129	37	(9)	1	[–5, 7]
Folate, µg *M* (*SD*) *M* [95% CI]	352	(1009	333	(106)	–7	[–109, 95]
Retinol, µg *M* (*SD*) *Mdn* (Range) *Mdn* [95% CI]	342	(109–17349)	342	(156)	107	[–30, 2414]
Beta carotene, µg *M* (*SD*) *M* [95% CI]	4569	(2056)	3895	[3044, 4984]^a^	761	[–1016, 2538]
Vitamin D, µg *M* (*SD*) *Mdn* (Range) *Mdn* [95% CI]	2.0	(0.6–15.7)	3.2	(1.8)	–0.6	[–0.9, 0.7]
Vitamin E, mg *M* (*SD*) *M* [95% CI] *Mdn* [95% CI]	8.8	(2.3)	10.5	[8.1, 13.6]^a^	–2.6	[–13.0, 0.3]
Tocopherols, mg *M* (*SD*) *M* [95% CI] *Mdn* [95% CI]	6.3	(2.9)	7.3	[5.1, 10.3]^a^	–2.3	[–12.2, 0.4]
Zinc, mg *M* (*SD*) *Mdn* [95% CI]	11	(4)	11	(2)	–1	[–2, 3]
Magnesium, mg *M* (*SD*) *M* [95% CI]	370	(146)	385	(85)	–33	[–156, 90]
Potassium, mg *M* (*SD*) *M* [95% CI]	3499	(10039	3448	(819)	–88	[–1035, 859]
Selenium, µg *M* (*SD*) *Mdn* [95% CI]	134	(63)	132	(43)	–21	[–44, 52]
Glycaemic Index, *M* (*SD*) *M* [95% CI]	51	(4)	51	(3)	1	[–2, 3]
Glycaemic Load, *M* (*SD*) *M* [95% CI]	116	(35)	123	(32)	–1	[–29, 28]

E%, percent energy from total macronutrient energy.

a Geometric mean.

Average nutrient intake per day at baseline. Estimated from 4-day weighed food records. Normally distributed variables are presented as *M* (*SD*). Transformed variables with normal distribution are presented as *Mdn* [95% CI]. Variables not normally distributed, neither before nor after transformation, are presented as *Mdn* (Range).

**Table 4.** Daily dietary intake from food groups at end of diet

Variable	Diabetes diet (N = 14)		Palaeolithic diet (N = 14)		Difference (N = 14)		*P*
Vegetables, g *M* (*SD*) *Mdn* (Range) *Mdn* [95% CI]	391	(93)	1150	(259–1430)	–724	[–854, –643]	.001
Vegetables, kJ *M* (*SD*) *Mdn* (Range) *Mdn* [95% CI]	503	(120)	1798	(291–2252)	–1346	[–1495, –1196]	.001
Fruits, g *Mdn* (Range) *M* [95% CI]	700	(333–792)	1403	(768–1635)	–733	[–890, –576]	< .001
Fruits, kJ *Mdn* (Range) *M* [95% CI]	1509	(758–1696)	5489	(2374–6243)	–3697	[–4428, –2967]	< .001
Meat, g *M* (*SD*) *Mdn* [95% CI]	84	(24)	242	(75)	–137	[–207, –117]	.001
Meat, kJ *M* (*SD*) *Mdn* [95% CI]	379	(140)	1061	(362)	–575	[–899, –479]	.001
Fish, g *M* (*SD*) *Mdn* (Range) *Mdn* [95% CI]	172	(100–190)	259	(58)	–107	[–127, –91]	.001
Fish, kJ *Mdn* (Range) *M* [95% CI]	716	(444–795)	929	(574–1009)	–193	[–280, –106]	.001
Eggs, g *M* (*SD*) *Mdn* (Range) *M* [95% CI]	43	(10)	26	(0–34)	20	[14, 27]	< .001
Eggs, kJ *M* (*SD*) *Mdn* (Range) *M* [95% CI]	253	(57)	152	(0–200)	121	[82, 160]	< .001
Cereals, g *Mdn* (Range) *Mdn* [95% CI]	317	(176–369)	0	(0–379)	305	[214, 323]	.001
Cereals, kJ *M* (*SD*) *Mdn* (Range) *Mdn* [95% CI]	3683	(697)	0	(0–4932)	3736	[2818, 4125]	.001
Legumes, g *Mdn* (Range) *Mdn* [95% CI]	44	(22–69)	6	(4–26)	38	[28, 46]	< .001
Legumes, kJ *M* (*SD*) *Mdn* (Range) *Mdn* [95% CI]	607	(304–950)	70	(10)	537	[393, 636]	< .001
Dairy products, g *Mdn* (Range) *Mdn* [95% CI]	422	(22–451)	0	(0–452)	413	[222, 424]	.002
Dairy products, kJ *Mdn* (Range) *Mdn* [95% CI]	909	(302–1375)	0	(0–1068)	909	[605, 1038]	.001
Sugars and cakes, g *Mdn* (Range) *Mdn* [95% CI]	1	(0–2)	12	(1–14)	–11	[–13, –6]	.001
Sugars and cakes, kJ *Mdn* (Range) *M* [95% CI]	12	(5–40)	158	(18–282)	–118	[–163, –73]	< .001
Oils and fats, g *M* (*SD*) *M* [95% CI]	32	(11)	19	(3)	13	[8, 19]	< .001
Oils and fats, kJ *M* (*SD*) *M* [95% CI]	1213	(411)	724	(111)	489	[292, 686]	< .001
Drinks, g *Mdn* (Range) *Mdn* [95% CI]	167	(110–446)	19	(17–141)	148	[126, 258]	.001
Drinks, kJ *Mdn* (Range) *Mdn* [95% CI]	39	(16–497)	48	(39–211)	–9	[–20, 53]	.680
Pre-cooked foods, g *Mdn* (Range) *Mdn* [95% CI]	0	(0–0)	0	(0–1)	0	[0, 0]	.110
Pre-cooked foods, kJ *Mdn* (Range) *Mdn* [95% CI]	0	(0–2)	0	(0–8)	0	[–3, 0]	.110
Snacks, g *Mdn* (Range) *Mdn* [95% CI]	3	(0–3)	17	(0–17)	–14	[–16, –12]	.001
Snacks, kJ *Mdn* (Range) *Mdn* [95% CI]	16	(0–16)	26	(0–26)	–10	[–18, –9]	.006
Sauces and dressings, g *Mdn* (Range) *Mdn* [95% CI]	7	(6–37)	11	(0–11)	–4	[–4, 9]	.400
Sauces and dressings, kJ *Mdn* (Range) *Mdn* [95% CI]	69	(46–447)	25	(1–31)	45	[43, 110]	.001

Average food eaten per day by weight and energy during a Palaeolithic and a diabetes diet. Estimated from 4-day weighed food records. Normally distributed variables are presented as *M* (*SD*). Transformed variables with normal distribution are presented as *Mdn* [95% CI]. Variables not normally distributed, neither before nor after transformation, are presented as *Mdn* (Range).

**Table 5.** Daily dietary nutrient intake at end of diet

Variable	Diabetes diet (N = 14)		Palaeolithic diet (N = 14)		Difference (N = 14)		*P*
Total weight, kg *M (SD) Mdn* (Range) *M* [95% CI]	2.5	(1.4–2.7)	3.1	(0.6)	–0.9	[–1.1, –0.7]	< .001
Total energy, MJ *Mdn* (Range) *Mdn* [95% CI]	10.4	(6.2–11.8)	10.9	(6.5–12.3)	–0.2	[–1.0, 0.0]	.048
Protein, g *M (SD) Mdn* (Range) *M* [95% CI]	103	(16)	120	(71–140)	–9	[–17, –1]	.030
Protein energy percent, E% *M (SD) Mdn* [95% CI]	18	(1)	18	(1)	0	[–1, 0]	.900
Carbohydrates, g *M (SD) Mdn* (Range) *M* [95% CI]	286	(52)	314	(188–358)	–14	[–31, 2]	.080
Carbohydrates energy percent, E% *Mdn* (Range) *M* [95% CI]	54	(48–57)	55	(51–56)	–1	[–3, 0]	.080
Fibre, g *M (SD) M* [95% CI]	47	(9)	62	(15)	–15	[–23, –7]	< .001
Fat, g *M (SD) M* [95% CI]	75	(19)	75	(17)	0	[–7, 6]	.900
Fat energy percent, E% *Mdn* (Range) *M* [95% CI]	28	[27, 30]^a^	26	(26–31)	2	[0, 3]	.100
Cholesterol, mg *M (SD) Mdn* (Range) *M* [95% CI]	326	(181–379)	314	(71)	–11	[–36, 14]	.400
Saturated fatty acids, g *M* [95% CI] *Mdn* [95% CI]	16	[13, 19]^a^	13	[11, 16]^a^	3	[1, 4]	.010
Monounsaturated fatty acids, g *M (SD) M* [95% CI]	36	(10)	36	(7)	0	[–3, 3]	.800
Polyunsaturated fatty acids, g *M (SD) Mdn* (Range) *M* [95% CI]	13	(8–14)	16	(4)	–4	[–5, –2]	< .001
Calcium, mg *M (SD) Mdn* (Range) *Mdn* [95% CI]	1086	(404–1206)	703	(166)	343	[194, 402]	.002
Iron, mg *M (SD) Mdn* (Range) *M* [95% CI]	22	(4)	22	(4)	–1	[–3, 1]	.500
Sodium, mg *M (SD) Mdn* (Range) *M* [95% CI]	1747	(587)	1024	(695–2558)	656	[324, 987]	.001
Vitamin A, µg *M (SD) Mdn* (Range) *Mdn* [95% CI]	1302	(654–1403)	3350	(1074)	–2251	[–2720, –1803]	.001
Vitamin B1, mg *M (SD) Mdn* (Range) *M* [95% CI]	2.3	(0.5)	2	(1–2)	0.3	[0.1, 0.44]	.004
Vitamin B2, mg *M (SD) Mdn* (Range) *M* [95% CI]	2.4	(1.1–2.7)	1.9	(0.4)	0.4	[0.2, 0.5]	.001
Folic acid, µg *M (SD) M* [95% CI]	431	(66)	558	(104)	–127	[–176, –78]	< .001
Vitamin C, mg *M (SD) Mdn* (Range) *Mdn* [95% CI]	219	(128–251)	472	(100)	–277	[–325, –244]	< .001
Vitamin B12, µg *M (SD) Mdn* [95% CI]	6.3	(1.6)	10.7	(2.6)	–4.1	[–05, –03]	< .001
Water, g *M (SD) Mdn* (Range) *M* [95% CI]	1609	(917–1746)	1972	(351)	–514	[–644, –384]	< .001
Alcohol, g *Mdn* (Range) *Mdn* [95% CI]	1.3	(0.5–6.8)	1.6	(1.3–3.2)	–0,3	[–01, 01]	.800
Glucose, g *Mdn* (Range) *Mdn* [95% CI]	18	(9–20)	83	(23–96)	–64	[–72, –44]	< .001
Fructose, g *Mdn* (Range) *Mdn* [95% CI]	30	(14–34)	86	(32–100)	–54	[–67, –41]	< .001
Saccharose, g *Mdn* (Range) *M* [95% CI]	27	(13–29)	66	(40–79)	–40	[–46, –33]	< .001
Myristic acid, g *Mdn* (Range) *M* [95% CI] *Mdn* [95% CI]	1.2	[1–1.6]^a^	0.5	(0.3–2.8)	0.7	[0.2, 1]	.020
Palmitic acid, g *M (SD) Mdn* [95% CI]	9.7	(2.7)	9.5	(3)	0.7	[–1.6, 1.3]	.800
Stearic acid, g *M (SD) M [95% CI] Mdn* [95% CI]	3.2	(1)	2.7	[2.1, 3.7]^a^	0.5	[–0.4, 1]	.020
Palmitoleic acid, g *M (SD) Mdn* [95% CI]	1.1	(0.3)	1.3	(0.3)	–0.1	[–0.2, 0.02]	.020
Oleic acid, g *M (SD) M* [95% CI]	34	(10)	35	(7)	0	[–3, 3]	.800
Linoleic acid, g *M (SD) Mdn* (Range) *M* [95% CI]	10	(7–11)	12	(3)	–2	[–3, –1]	< .001
Alpha-linolenic acid, g *M (SD) Mdn* [95% CI]	0.9	(0.2)	1.7	(0.4)	–0.8	[–0.9, –0.6]	< .001
Arachidonic acid, g *M (SD) Mdn* (Range) *M* [95% CI]	0.1	(0.1–0.2)	0.3	(0.1)	–0.2	[–0.2, –0.1]	< .001
Niacin, mg *Mdn* (Range) *M* [95% CI]	52	(31–58)	64	(36–76)	–13	[–18, –7]	< .001
Folate, µg *M (SD) M* [95% CI]	431	(66)	553	(110)	–121	[–176, –67]	< .001
Retinol, µg *M (SD) M* [95% CI]	246	(70)	103	[78, 134]^a^	133	[94, 171]	< .001
Beta carotene, µg *M (SD) Mdn* (Range) *Mdn* [95% CI]	5362	(2466–5580)	17370	(5836)	–13450	[–15857, –10728]	.001
Vitamin D, µg *M (SD) Mdn* (Range) *M* [95% CI]	5.2	(1)	5.5	(3–6)	0.2	[–0.3, 0.7]	.300
Vitamin E, mg *M (SD) Mdn* (Range) *M* [95% CI]	15	(10–17)	26	(6)	–12	[–15, –9]	< .001
Tocopherols, mg *Mdn* (Range) *M* [95% CI]	13	(8–15)	22	(13–25)	–9	[–11, –6]	< .001
Zinc, mg *M (SD) Mdn* [95% CI]	14	(3)	12	(3)	3	[0, 3]	.020
Magnesium, mg *Mdn* (Range) *M* [95% CI]	632	(413–703)	563	(116)	10	[–45, 65]	.700
Potassium, mg *M (SD) Mdn* (Range) *M* [95% CI]	4300	(2536–4681)	7487	(1671)	–3626	[–4410, –2841]	< .001
Selenium, µg *Mdn* (Range) *M* [95% CI]	182	(97–200)	157	(92–175)	17	[8, 26]	.001
Glycaemic Index, *M (SD) M* [95% CI]	50	(2)	50	(1)	0	[–1, 1]	.600
Glycaemic load, *Mdn* (Range) *M* [95% CI]	152	(91–178)	159	(91–179)	–8	[–17, 1]	.100

E%, percent energy from total macronutrient energy.

a Geometric mean.

Average nutrient intake per day during a Palaeolithic and a diabetes diet. Estimated from 4-day weighed food records. Normally distributed variables are presented as *M* (*SD*). Transformed variables with normal distribution are presented as *Mdn* [95% CI]. Variables not normally distributed, neither before nor after transformation, are presented as *Mdn* (Range).

## Discussion

There were no differences between the two diets in effects on the primary outcomes HbA1c and fructosamine. Body weight was kept stable, and the contents of the two diets differed, as planned, regarding the intake of the food groups cereal grains, dairy products, and legumes, and the diets were successfully matched for macronutrient composition and glycaemic load but not for fibre content. These results suggest that characteristic food group differences and accompanying fibre content differences between a Palaeolithic and a diabetes diet do not cause an effect on glycaemic control when body weight is kept stable and macronutrient composition and glycaemic load are matched. Notably, glucose and fibre intake were both higher in the Palaeolithic diet compared with the diabetes diet, possibly counteracting each other for glycaemic control effects. However, as discussed in detail below, the maintenance of stable body weight may have been the primary factor underlying the lack of difference in glycaemic control between diets.

Previous randomised controlled trials in weight-stable participants with type 2 diabetes found that 5–6 weeks of low-carbohydrate/high-protein diets improve glycaemic control compared with a diabetes diet.^(27–29)^ On the contrary, trials 6 months or longer comparing low-carbohydrate content or low glycaemic index diets with a diabetes diet did not find an improvement in glycaemic control.^(30–33)^ A systematic review of trials lasting 6 months or longer also found no effect of macronutrient composition on glycaemic control in overweight and obese adults with type 2 diabetes, provided there were no differences in weight loss between the groups.^(5)^ When reported and not matched for, fibre content, and glycaemic load usually also differed between diets in these studies. Taken together, the results from these previous studies indicate that macronutrient composition, fibre content and glycaemic load may not affect glycaemic control beyond weight loss, at least in the long term.

Interestingly, in the current study, fructosamine and glycated albumin were similarly improved by both diets. This result could indicate that glycaemic control can improve from short-term dietary interventions without simultaneous reduction in body weight.

Fructose and glucose intake was three- to five-fold higher in the Palaeolithic diet compared with both baseline and the diabetes diet. The differences stem from the need to match diets in terms of carbohydrate content and replacing non-Palaeolithic starchy foods such as cereal grains with non-starchy carbohydrate sources such as fruits and dried fruits. Glycaemic control improved during both diets, despite the higher intake of fructose and glucose in the Palaeolithic diet. This finding is congruent with previous results on the beneficial effects of high fruit intake on insulin sensitivity,^(10,11)^ the reduction in AGEs consumption,^(14)^ and improved glycaemic control.^(34)^ The improved glycaemic control during both diets could be caused by positive expectations among participants of the supposedly healthy intervention diets.^(35)^

There were no differences between diets in effects on blood lipids. However, the decrease in total cholesterol and LDL during the Palaeolithic diet and the decrease in HDL and increase in triglyceride during the diabetes diet suggest a more beneficial effect on the blood lipid profile from the Palaeolithic diet than from the diabetes diet. The only difference between diets concerning a change of an outcome variable during diet was for biceps skinfold, with a decrease during the Palaeolithic diet and an increase during the diabetes diet. However, this result should be interpreted with caution, as there were no corresponding changes in other measures of skinfolds or body fat, which also is less trustworthy since only data from the first intervention period could be used due to a carry-over effect.

Reported dietary intakes during interventions, with, as planned, resulting differences between diets in contents regarding the food groups cereal grains, dairy products, and legumes, indicate an overall compliance with the dietary interventions. As observed in previous studies on the Palaeolithic diet, the reported calcium intake of 703 mg/day from the Palaeolithic diet was below the recommendation of 800 mg/day from the Swedish Food Agency,^(36)^ which is most likely due to the exclusion of dairy products. Systematic reviews and meta-analyses suggest that, in epidemiological studies, increasing dietary calcium intake from low levels to around 750 mg/d is associated with a decreased risk of type 2 diabetes in epidemiological studies;^(37)^ however, this association is not supported by findings from intervention studies.^(38)^ Therefore, it is unlikely that the lower calcium intake in the Palaeolithic diet would have affected glycaemic control. Regarding other micronutrients intake, there were significant differences between diets at the end of the interventions, with certain micronutrients being higher after the Palaeolithic diet and others after the diabetes diet. However, besides the mentioned exception of calcium, the daily intake of all vitamins and minerals was well above the recommendations from the Swedish National Food Agency. Given the lack of evidence supporting the benefits of vitamin or mineral supplementation in subjects with type 2 diabetes without underlying deficiencies,^(39)^ it is unlikely that these differences might have influenced the results.

The differences in glucose, fructose, and saccharose intake between the Palaeolithic and the diabetes diets did not translate into a difference in glycaemic control, which is in line with a meta-analysis concluding that most food sources of fructose-containing sugars (especially fruit) do not negatively impact glycaemic control when substituted for macronutrients in isocaloric conditions.^(40)^

### Strengths

Our aims of keeping participants’ body weight stable, as well as matching macronutrient composition and glycaemic load between the two diets, and accounting for diabetes medications were all achieved, thereby negating accompanying differences in effects on glycaemic control when comparing diets. Furthermore, daily lunch was provided to all participants during the diets to increase compliance. To decrease the risk of bias, great care was given to present both study diets as equally favourable.

### Limitations

A major limitation comes from not achieving our aim of keeping fibre content the same in both diets, thereby precluding the possibility of negating accompanying differences in effects when comparing diets. Fibre content possibly ended up differing between diets because participants did not consume or properly report consumption of fibre added in the diabetes diet in order to compensate for an expected lower fibre intake compared with the Palaeolithic caused by greater amounts of fruit and vegetables in the latter. Despite an average increase of reported energy intake of approximately 2 MJ per day during both dietary interventions compared with baseline, there was a non-significant decrease in body weight. The discrepancy indicates a relative under-reporting of dietary energy intake at baseline or over-reporting during the intervention. Future study instructions should put more emphasis on the importance of accurately reporting the consumption of food.

Another limitation comes from the short duration of the study. The power calculation was based on an only 2-week-long trial which still managed to find a trend toward greater reduction in fructosamine from the Palaeolithic diet.^(8)^ This trial, with twice as long 4-week interventions, was therefore considered to be long enough to observe a significant difference in glycaemic control between interventions. This was deemed to be likely, especially since the intervention strength was estimated to increase by providing participants with daily lunch in the hospital kitchen, which, for logistical reasons, was possible only for a maximum of two periods of 4 weeks each.

## Conclusions

There were no differences between the two diets in effects on the primary outcomes HbA1c and fructosamine. Body weight was kept stable, and the contents of the two diets differed, as planned, regarding the food groups cereal grains, dairy products and legumes, and the diets were successfully matched for macronutrient composition and glycaemic load but not for fibre content. These results suggest that characteristic food group differences and accompanying fibre content differences between a Palaeolithic and a diabetes diet do not cause an effect on glycaemic control when body weight is kept stable and macronutrient composition and glycaemic load are matched.

## Supporting information

Fontes-Villalba et al. supplementary material Fontes-Villalba et al. supplementary material

## Data Availability

The dataset analysed during the current study is available from the corresponding author upon reasonable request.

## Supplementary material

The supplementary material for this article can be found at https://doi.org/10.1017/jns.2025.10028
