# Supplementary material for: Randomised crossover controlled trial of dietary interventions for glycaemic control when body weight is kept stable
Source: J Nutr Sci. 2025 Aug 29;14:e59. doi: 10.1017/jns.2025.10028 (PMC12418275; doi:10.1017/jns.2025.10028)
Supplement: Fontes-Villalba et al. supplementary material [file S2048679025100281sup001.pdf]

Supplementary table 1

Adjustments of Recommended Daily Dietary Energy Intake During Diet to Maintain Stable Body Weight

Variable	Diabetes diet		Paleolithic diet		Difference	
Recommended daily dietary energy intake at start of diet, MJ M (SD) M [95% CI]	9.1	(1.7)	9.3	(1.6)	-0.2	[-1.3, 0.9]
Adjustment of recommended daily dietary energy intake during study, MJ M (SD) Mdn (Range) M [95% CI]	0.5	(1.5)	0.0	(0.0 to 2.5)	-0.3	[-1.6, 0.9]
Relative adjustment of recommended daily dietary energy intake during study, % M (SD) Mdn (Range) M [95% CI]	6.0	-16	0.0	(0.0 to 27)	-2.7	[-16, 11]
Recommended daily dietary energy intake at end of diet, MJ Mdn (Range) Mdn [95% CI]	10.5	(6.1 to 11.7)	10.5	(6.1 to 11.7)	0.0	[-1.0, 0.0]
Type of adjustment of recommended dietary energy intake during study decreased/unchanged/increased, n (%)	2 (14%) / 5 (36%) / 7 (50%)		0 (0%) / 8 (57%) / 6 (43%)			
Weeks on diet when recommended dietary energy intake was adjusted, M (SD) Mdn (Range) M [95% CI]	2.0	(1.0 to 3.0)	2.0	(0.7)	-0.2	[-4.5, 4.2]
Adjustments of recommended daily dietary energy intake during study per participant, n Mdn (Range) Mdn [95% CI]	1.0	(0.0 to 2.0)	0.0	(0.0 to 2.0)	0.5	[-0.5, 1.0]
